# Improving Computer-Aided Thoracic Disease Diagnosis through Comparative Analysis Using Chest X-ray Images Taken at Different Times

**DOI:** 10.3390/s24051478

**Published:** 2024-02-24

**Authors:** Sung-Nien Yu, Meng-Chin Chiu, Yu Ping Chang, Chi-Yen Liang, Wei Chen

**Affiliations:** 1 Department of Electrical Engineering, National Chung Cheng University, Chiayi County 621301, Taiwan; ab30310@gmail.com (M.-C.C.); joe.yuping@gmail.com (Y.P.C.); 2 Center for Innovative Research on Aging Society (CIRAS), National Chung Cheng University, Chiayi County 621301, Taiwan; 3 Division of Pulmonary and Critical Care Medicine, Chiayi Christian Hospital, Chiayi County 600566, Taiwan; 07125@cych.org.tw

**Keywords:** lung disease, chest X-ray image, image segmentation, image alignment, deep learning

## Abstract

Medical professionals in thoracic medicine routinely analyze chest X-ray images, often comparing pairs of images taken at different times to detect lesions or anomalies in patients. This research aims to design a computer-aided diagnosis system that enhances the efficiency of thoracic physicians in comparing and diagnosing X-ray images, ultimately reducing misjudgments. The proposed system encompasses four key components: segmentation, alignment, comparison, and classification of lung X-ray images. Utilizing a public NIH Chest X-ray14 dataset and a local dataset gathered by the Chiayi Christian Hospital in Taiwan, the efficacy of both the traditional methods and deep-learning methods were compared. Experimental results indicate that, in both the segmentation and alignment stages, the deep-learning method outperforms the traditional method, achieving higher average IoU, detection rates, and significantly reduced processing time. In the comparison stage, we designed nonlinear transfer functions to highlight the differences between pre- and post-images through heat maps. In the classification stage, single-input and dual-input network architectures were proposed. The inclusion of difference information in single-input networks enhances AUC by approximately 1%, and dual-input networks achieve a 1.2–1.4% AUC increase, underscoring the importance of difference images in lung disease identification and classification based on chest X-ray images. While the proposed system is still in its early stages and far from clinical application, the results demonstrate potential steps forward in the development of a comprehensive computer-aided diagnostic system for comparative analysis of chest X-ray images.

## 1. Introduction

Lung-related illnesses are prevalent health concerns among the human population, with lung cancer standing out as a particularly lethal condition [1]. In the domain of thoracic medicine, healthcare professionals routinely undertake the arduous task of examining and comparing numerous X-ray images to monitor the development of lesions. This process is not only time-consuming but can also induce fatigue, potentially leading to human errors or misjudgments [2].

With the rapid advancements in the computer industry, image-processing technologies have gained extensive utilization in the analysis of medical images. These applications include image enhancement, image restoration, and the segmentation of organ areas, all of which aid in achieving more precise disease diagnoses [3,4]. Furthermore, recent developments in artificial intelligence technology have also seen applications in the domain of medical imaging analysis.

Given these advantages, our aim is to harness these capabilities to create a computer-aided diagnostic system for the comparative analysis of two thoracic X-ray images of the same patient taken at different points in time, highlighting the discrepancies between them. The objective is to enable the detection of disease progression and anomalous areas, thus facilitating the physician’s task of pinpointing and closely examining the areas that may be cause for concern, enhancing diagnostic efficiency, and reducing the likelihood of misinterpretation.

However, the study of the comparative analysis of chest X-ray images taken at different times has not been widely explored in the literature. There are few datasets that contain chest X-ray images taken at different times from the same patient. As a result, we created such a dataset by sampling from the public NIH Chest-Xray14 dataset, which contains 112,120 X-ray images acquired from 30,805 patients. We have to select only a very small subset of samples that form pairs of images from the same patient, greatly limiting the number of samples available for training and testing. In an attempt to substantiate the comparative discussion, we further employed a local dataset gathered by the Chiayi Christian Hospital in Taiwan that purposely gathered pairs of chest X-ray images from 30 patients at different time points to facilitate the comparative analysis.

A four-stage framework was proposed to accomplish this task, including the lung segmentation, registration, comparison, and disease classification stages. As the study of comparative analysis in chest X-ray images was new, we employed both traditional machine-learning techniques and deep-learning approaches to provide a more comprehensive discussion. A deep-learning network was further employed in disease classification, where we explored whether the differential information between the pre- and post-images could contribute to the accuracy of disease classification.

The contribution of this study can be summarized below:

1. Proposed a computer-aided diagnostic system that integrates segmentation, registration, comparison, and classification of lung X-ray images into one framework
2. Compared traditional and deep-learning methods in each stage in terms of performance metrics and speed.
3. Propose the inclusion of differential information in disease classification and show improved results.

The following sections will be organized as follows. Section 2 describes related works in the areas of image segmentation, registration, and classification. Section 3 details the methodology utilized in each of the four stages. Section 4 presents and discusses the experimental designs and findings. Finally, Section 5 concludes the study.

## 2. Related Works

### 2.1. Lung Image Segmentation

Traditional segmentation techniques for chest X-ray images make use of image-processing methods, including image enhancement, thresholding, and morphological operations, to identify and extract the lung field area. A commonly employed image enhancement technique is histogram equalization [5], which adjusts the distribution of intensity values in the image’s histogram to enhance the overall image contrast. In 2001, Li et al. [6] introduced a lung-segmentation approach based on histogram statistics to identify the lung’s location and delineate it within the image. In 2006, Cheng [7] proposed a lung-segmentation technique that relied on grayscale thresholds. This method involved dividing the chest X-ray image into three regions and utilizing the cumulative histogram to pinpoint the left lung, right lung, and the boundaries of the two lung lobes. Chen et al. [8] employed morphological operators for edge detection and connected region labeling to achieve lung segmentation.

In contrast, more contemporary image segmentation methods are frequently integrated with deep-learning networks, particularly convolutional neural networks (CNNs). These methods are typically rooted in the concept of object detection. Mainstream object detection methods fall into two primary categories. The first category involves network architectures that employ a two-stage approach for object detection, exemplified by methods like RCNN [9], Fast RCNN [10], and the Faster RCNN series [11]. The second category consists of network architectures that utilize a one-stage approach for object detection, such as SSD [12] and the YOLO series [13].

### 2.2. Lung Image Registration

Image registration is a fundamental image-processing technique used to align two images obtained under different conditions. This may include images from different capture equipment, varying shooting angles, or at different time points. Image registration involves employing a spatial transformation to map one image onto another, achieving alignment and matching. It is typically viewed as an optimization task and finds applications across medical and nonmedical domains [14,15]. Intensity-based image registration relies on the intensity values of the images to align and transform them. An image comprises a set of discrete intensity values represented as grids. The transformation function maps points from the original image to the coordinate system of the target image, and the algorithm iteratively optimizes this mapping. As this transformation occurs in the continuous domain, interpolation methods are employed in the process. Several registration methods have been discussed in the works of Fitzpatrick et al. [16], Yaniv [17], and Goshtasby [18].

In recent years, techniques that combine convolutional neural networks and image registration have emerged. For instance, B. de Vos et al. [19] and H. Li, Y. Fan et al. [20] have proposed unsupervised architectures, integrating convolutional neural networks with spatial transformation networks [21] to register pairs of images. However, these methods are preliminary and come with certain limitations, primarily restricted to handling minor transformations in specific regions.

### 2.3. Lung Image Classification

The utilization of deep learning on chest X-rays for lung-related disease classification has been widely studied, especially in recent years, due to interest in the efficient detection of COVID-19 [22]. The application of various well-known models such as VGG [23], Resnet [24,25], Inception [26], Mobilenet [27] and DenseNet [28,29,30], as well as models incorporating attention [31], pretraining [32] and ensembling [33] have been studied. Many have shown that segmenting the lung area before classification was able to improve model performance [34,35]. However, to the best of our knowledge, the use of differential information between X-ray images taken from the same patient at different times in lung disease classification has not been explored.

## 3. Methodology

### 3.1. Chest X-ray Datasets

Two distinct image datasets were employed in this study. The first dataset utilized was the NIH Chest X-ray14 dataset [36], a compilation released by the National Institutes of Health (NIH) (Bethesda, MD, USA). The second dataset comprised 30 pairs of chest X-ray images gathered by the Chiayi Christian Hospital (CYCH) in Chiayi, Taiwan (CYCH-IRB No. IRB2019007). Each pair of images originated from the same patients, captured at different time points.

The NIH database includes a total of 112,120 X-ray images acquired from 30,805 patients. These 1024×1024 pixels images encompass various shooting techniques, including the posteroanterior view and anteroposterior view. The database features 15 categories, comprising 14 disease categories along with one “no findings” category. The labeling of images in this dataset was carried out by applying natural language processing technology on the medical reports to extract the labels. The estimated label accuracy exceeds 90% and thus is suitable for weakly supervised learning. It is worth noting that the original medical reports tied to this database have not been officially published. For more comprehensive insights into the labeling process, one may refer to Wang et al.’s article [37].

As this study focuses on the comparative analysis of chest X-ray images from the same patient taken at different times, we selected samples from the NIH dataset that has at least one other image from the same patient to form pairs of pre- and post-images. Only a very small subset of 3200 pairs of pre- and post-image was found, which may adversely impact model learning and generalizability. Moreover, due to the substantially lower image numbers for two disease categories (Edema and Hernia) compared to the others, these two categories of image pairs were excluded. As a result, 3188 image pairs encompassing 12 categories of lung diseases and a “no findings” category were employed for analysis. We note that the lack of image pairs to facilitate the training of deep-learning models is a limitation of this study.

We summarize the selected dataset in Table 1. Please note that one sample may contain multiple diseases, and thus, the total number of pairs in the dataset is less than the sum of diseases.

Given the extensive volume of images contained within the NIH database and the diversity of conditions experienced by each patient, some images are subject to variations in quality, resulting in a subset of images that are less clear and complete. A data cleaning process was applied to exclude images with excessive blurriness or the presence of implanted medical devices and wiring, including pacemakers and wires, to ensure the quality of the dataset for model learning. The NIH database is utilized in the lung image segmentation, registration, and classification stages of the experiment.

The second image dataset is a private dataset comprising 30 pairs of X-ray images gathered by the Chiayi Christian Hospital (CYCH). This dataset purposely collected X-ray images of the same patient taken at different times to facilitate the experiments and discussions in comparative analysis, and thus will only be utilized during the testing phase of lung image registration and comparison stages of the experiment.

### 3.2. The Framework

**Figure 1:**
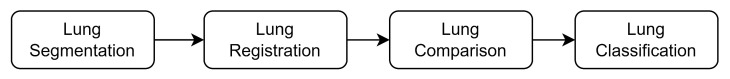
Experimental flowchart.

The experimental framework was structured into four distinct stages, outlined in Figure 1.

1. Lung Segmentation Stage : In the first stage, the primary focus was on segmenting the lung areas within the images. This segmentation was crucial to ensure that subsequent processing concentrated on the lung region rather than the entire image. Two approaches were employed in this stage: a traditional lung-segmentation method and a deep learning-based lung-segmentation method. The performance of these two methods was rigorously compared.
2. Lung Registration Stage: The second stage performed registration of lung images. In this phase, the contour of the lung area in the original image was aligned and deformed to fit the structure of the target image. The original image corresponded to the X-ray image captured at the a priori time, while the target image represented the X-ray image obtained at a later time. The objective here was to ensure that subsequent lung image comparisons were based on aligned lung areas. This approach aided in detecting real changes in organ-related or malignant tissues, as opposed to misalignment artifacts. Similar to the segmentation stage, this stage also featured the development and comparison of both traditional and deep learning-based registration methods.
3. Lung Comparison Stage: In the third stage, a specific formula was utilized to quantify the difference between the pre- and post-images. Nonlinear transformations were applied to accentuate these differences, and they were visualized as heat maps overlaid on the images. The objective was to facilitate quick comparisons of two images taken at different times by physicians for disease progression diagnosis. The difference image is also subsequently utilized in the classification network to assess its contribution to the overall classification performance.
4. Lung Classification Stage: The fourth and final stage involved the classification of lung images afflicted by various thoracic diseases. Deep-learning networks were employed for the classification tasks. To evaluate the contribution of the difference image in classification, two approaches were proposed. The first, a single-input network architecture, performed classification based on a single image, which could be either the original image or the difference image synthesized from the previous stage. In comparison, the second, a dual-input network architecture, conducted classification using both the original and the difference images. The performance of these two classifier architectures was systematically compared to determine their relative efficacy.

The following subsections describe each stage in greater detail.

### 3.3. Lung Image Segmentation

The segmentation of the lung area constituted the first phase of this study. The objective was to identify and delineate the region of interest (ROI), specifically the lung area, enabling subsequent analysis and processing. We compare two distinct lung-segmentation methodologies: one rooted in traditional image processing and the other based on deep-learning techniques.

#### 3.3.1. Traditional Lung-Segmentation Method

**Figure 2:**
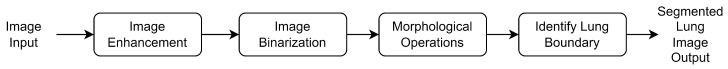
Flow chart of traditional lung-segmentation method.

The traditional segmentation method adopted in this study includes four procedures, namely image enhancement, image binarization, morphological operations, and edge detection, as shown in Figure 2. The ultimate goal was to detect the boundaries of the lung area.

The enhancement of X-ray images involved the implementation of the Contrast Limited Adaptive Histogram Equalization (CLAHE) technique [5,38]. Subsequently, Otsu’s image binarization method [39] was applied to categorize image pixels into black or white. Pixels with original values above the threshold were set to 255 (white), while those below the threshold were assigned a value of 0 (black).

Although the image binarization method effectively segregated the lung area from the background, it may have retained some undesired artifacts and noise. To mitigate these issues, the Connected-Component Labeling technique [40] was employed, facilitating the identification and subsequent removal of connected regions in binary digital images that are not the lung area. First, connected regions related to the background were removed. Then, the “open” and “close” morphological operations [41] were applied to yield a smoother representation of the lung area. Finally, to eliminate the small sporadic noise areas, we calculated the areas of each connected region and removed regions with areas smaller than the mean.

Once the precise coordinates defining the lung area were established, they were mapped back to the original image. The top, bottom, left, and right boundaries of the lung area were determined from the segmented binary image, and, adding a small allowance, the lung ROI was determined and cropped from the original image.

#### 3.3.2. Deep-Learning Lung-Segmentation Method

The objective of this stage was the precise detection of lung areas. Given the clarity of the target and the relatively large area to be identified, there was no necessity for an excessively complex or deeply layered network architecture. To accomplish this task, a single-stage object detection network named RetinaNet [42] was employed in this research.

RetinaNet [42] adopts a Residual Network (ResNet) [43] as its backbone, and it leverages the Feature Pyramid Network (FPN) [44] architecture to extract multiscale feature maps, which were then passed to the network’s class and box subnets for classification and bounding box regression. In an effort to enhance computational efficiency, we substituted the backbone network with a lighter-weight 18-layer residual network (ResNet18) while keeping other essential parameters unchanged.

The hyperparameters used for training the RetinaNet for lung-area detection are summarized in Table 2. The initial learning rate was set to 0.0001. During the training process, a “patience” parameter was employed, which reduces the learning rate by a decay factor if the performance did not improve after “patience” consecutive evaluations. In this case, the “patience” index was set to 3, and the learning rate decay factor was set to 0.1. Other parameters included a batch size of 1, a total of 200 training epochs, and the utilization of the Adam optimizer.

### 3.4. Lung Image Registration

In this stage, we compared two lung-area registration algorithms: one based on traditional image processing and the other utilizing deep-learning networks. The dataset employed in this stage consisted of 3200 pairs of pre- and post-images, carefully selected from the aforementioned NIH database. Before proceeding with lung-area registration, the lung region was first segmented from the entire image and uniformly adjusted to a consistent image size of 512×512. The post-image served as the reference image, while the pre-image was designated as the working image. Histogram matching was first performed on the working image before applying the registration algorithms to enhance efficiency.

#### 3.4.1. Image Histogram Matching

Histogram matching [45] is an image-processing technique that involves the calculation of the probability density function of grayscale values in an image and then matching it to a specified target image. This matching process ensures that the histogram distribution of the original image becomes similar to that of the specified reference image. Consequently, it can function as a calibration technique, enabling the adjustment of brightness disparities between different detectors. In this study, the histogram matching algorithm [45] was employed to calibrate the histogram distributions of the working image to the reference image, therefore facilitating subsequent image registration tasks.

#### 3.4.2. Traditional Lung Image Registration Method

The traditional lung image registration method employed in this study was implemented using the image registration library provided by the open-source SimpleITK toolkit [46]. SimpleITK is a research project developed collaboratively by the National Library of Medicine, Mayo Clinic, Kitware, and the University of Iowa. Since 2019, the development of SimpleITK has received support from the National Institute of Allergy and Infectious Diseases (Bethesda, MD, USA).

Using the toolkit for image registration requires specifying four critical components: transform, similarity metric, interpolator, and optimizer. In this study:

- Transform: We employed the displacement field transform, which is a dense deformable transform suitable for 2D or 3D coordinates space.
- Similarity metric: The similarity metrics selected are the Advanced Normalization Tools(ANTS) neighborhood correlation and the joint histogram mutual information to assess the similarity between the two images.
- Interpolator: The interpolator used is the default linear interpolation method to interpolate pixel values.
- Optimizer: The optimizer utilized is the gradient descent method.

For specific usage instructions and examples, please refer to the registration usage example provided by SimpleITK [47].

#### 3.4.3. Deep-Learning Lung Image Registration Method

In this study, the deep-learning image registration algorithm adopted was VoxelMorph [48]. VoxelMorph is an image registration network that employs U-Net [49,50] to generate displacement fields and spatial transformations to create aligned images. Two network architectures are available for generating the displacement field: VoxelMorph-1 and VoxelMorph-2. In this study, VoxelMorph-2 was chosen due to its deeper network layers, which enable it to capture more information and exhibit greater robustness during training. Other parameters were kept consistent with the literature and were not altered.

The architecture of VoxelMorph-2 employs the U-Net as its fundamental structure, which comprises an Encoder and a Decoder. During the encoding phase, multiple convolution operations are applied, reducing the feature map size by half after each operation until it reaches 1/16 of the original input image size. In the decoding phase, upsampling convolutions are used to restore the feature map to the original input image size. At certain layers, the feature map in the decoding stage is concatenated with the corresponding layer in the encoding stage, allowing it to learn the encoding features. The Leaky ReLU serves as the activation function in both the encoding and decoding stages.

The hyperparameters employed for training the VexelMorph-2 for lung-area registration are detailed in Table 3. These include a learning rate of 0.0001, a batch size of 8, an epoch number of 400, and the utilization of the Adam optimizer.

### 3.5. Lung Image Comparison

During the lung image comparison stage, we computed the intensity changes in the registered lung images and highlighted the differing areas using heatmaps. The primary objective was to facilitate quick comparisons of two images taken at different times by physicians for disease progression diagnosis. Furthermore, nonlinear conversion functions were applied to emphasize less apparent differences.

In this study, the difference in pixel values between the pre- (fixed) and post- (moving) images was utilized to accentuate varying regions. The pixel values in both pre-image $Img_{1}$ and post-image $Img_{2}$ were initially normalized to the 0 to 1 range. Subsequently, the two aligned images were subtracted, and absolute difference values were computed. These values were then multiplied by the pixel values of the post-image $Img_{2}$ to emphasize changes, as demonstrated in Equation (1), where *D* is the difference image:

(1) $$D=Img_{2}\times \left|Img_{2}-Img_{1}\right|$$

**Figure 3:**
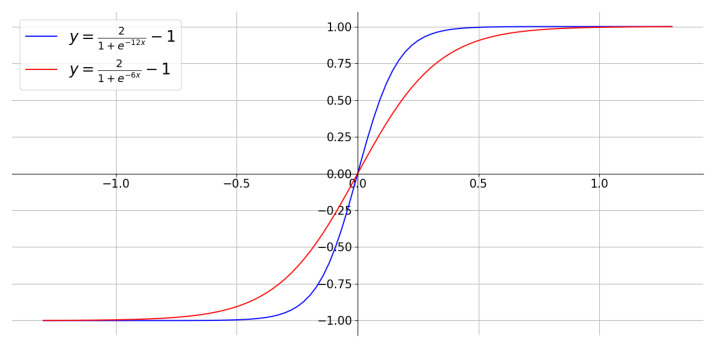
Nonlinear transformation functions.

In some cases, there may be regions with minute differences between the two images. To accentuate these differences, we proposed using Equations (2) and (3) as nonlinear transformations, where $D^{*}$ is the enhanced difference image. The transformations were sigmoid-like functions that squeeze the input values to within −1 to 1 but utilizing only the positive range. The transformation is designed so that difference values close to zero are still close to zero, but larger differences would be enhanced without exceeding 1 to keep the values within the image grayscale range. The coefficient in the exponential alters the extent of curvature, thus allowing control of the amount of enhancement as illustrated in Figure 3.

Finally, the post-image and the non-linearly transformed difference map were combined into the final highlighted image $Img_{new}$, as depicted in Equation (4). This highlighted image was employed in subsequent classification experiments to assess whether the inclusion of difference information improved classification results.

(2) $$D^{*}=f\left(D\right)=\frac{2}{1+e^{-6D}}-1$$

(3) $$D^{*}=f\left(D\right)=\frac{2}{1+e^{-12D}}-1$$

(4) $$Img_{new}=Img_{2}+D^{*}\left(\mathrm{or}\;D\right)$$

Human vision is more adept at detecting minor changes in a color map compared to grayscale intensity. Consequently, we opted to use a color heat map to depict the differences between the images. After computing the variance in pixel values, different colors were employed to denote the levels of difference based on human visual perception. In this representation, red signifies areas with greater differences, while blue corresponds to those with smaller differences. To achieve the heat map presentation, this study made use of the colormap library provided by OpenCV.

### 3.6. Lung Image Classification

We devised two network architectures based on the DenseNet [51], namely a single-input architecture and a dual-input architecture. The single-input architecture classifier takes in either the original image or the synthesized difference image from the comparison stage, while the dual-input architecture classifier utilizes information from both the original and difference images. The input images were subjected to lung image segmentation and resized to a uniform size of 512×512 for input into the classification networks. Experiments were designed to validate the contributions of the difference images in enhancing classifier performance.

#### 3.6.1. Single-Input Network

**Figure 4:**
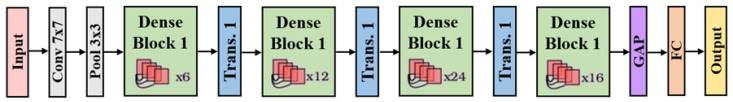
Single-input network architecture.

In this study, the single-input network architecture employed is the Dense Convolutional Network with 121 layers (DenseNet121) [51] with minor modifications. This network comprises 4 dense blocks and 3 transition layers, as illustrated in Figure 4. The last dense block contains 16 channels, which undergo a Global Average Pooling layer to produce a 16-dimensional feature vector. This feature vector is then fed into a fully connected layer with the sigmoid activation function to produce the final multi-label classification result. The single-input image can be the post-image or the synthesized difference image. Classification results based on different input types are compared.

#### 3.6.2. Dual-Input Network

**Figure 5:**
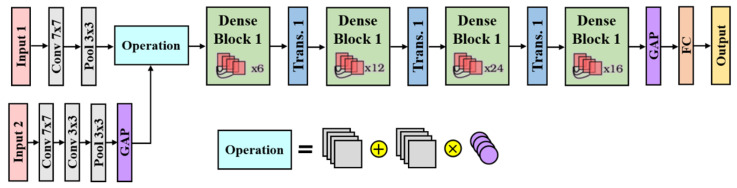
Dual-input network architecture.

To evaluate the contribution of the difference image in enhancing classifier performance, a dual-input network architecture is formulated for the classification tasks. The structure is depicted in Figure 5.

The dual-input network architecture consists of two inputs: Input 1 represents the post-image, and Input 2 represents the synthesized difference image. Input 1 undergoes processing through a convolution layer followed by a pooling layer, similar to its single-input network counterpart. On the other hand, Input 2 is processed using two convolution layers: a pooling layer and a Global Average Pooling (GAP) layer. The GAP layer calculates the average value of the feature map after the pooling layer, providing a weighting value to be applied on each channel from input 1. The information from these two images is then integrated using the “operation” block. The “operation” block enhances the feature map from Input 1 by multiplying it with (1 + weighting from Input 2) and then feeds the result into the subsequent network for classification. Experiments were devised to compare the classification results with or without the difference image as additional information for the classifiers. The classification outcomes obtained based on the single-input and dual-input networks were compared and validated.

The hyperparameters used to train the two networks are detailed in Table 4.

## 4. Experiments and Results

In this section, we detail the experimental designs and results of each stage and compare traditional and deep-learning methods. We provide both qualitative and quantitative evaluations. The experiments were tested using the hardware specifications detailed in Table 5. The software environment utilized in this study was Python 3.6, and the deep-learning networks were built using PyTorch [52].

### 4.1. Lung-Segmentation Experiments and Results

This study utilized two lung-area segmentation methods, one employing traditional image-processing techniques and the other utilizing deep learning. The former method involved typical image enhancement, binarization, and morphology calculations to segment lung areas. The latter method utilized the RetinaNet object detection network for lung-area detection. A set of 2000 chest X-ray images from the NIH database was selected for training and validation. The original images were resized to dimensions of 512×512 for computational efficiency. The segmented lung area would be remapped to its original image size after acquiring the coordinates of the lung region. The ground truth lung-area labeling was manually annotated by us to train the RetinaNet in a supervised fashion.

#### 4.1.1. Evaluation Methods

**Figure 6:**
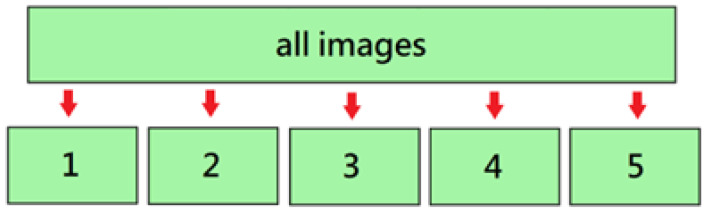
Schematic diagram of image data split.

(1) Traditional Lung-Segmentation Test Method:

The test method employed here involves dividing all the image data into five equal parts. As illustrated in Figure 6, with a total of 2000 images, each part contains 400 images. After testing each dataset, we obtained five groups of results, and these results were then averaged. This test method is designed to be comparable with the 5-fold cross-validation method performed for the deep-learning method but without the training phase, as that is not required for the traditional lung-segmentation method.

**Figure 7:**
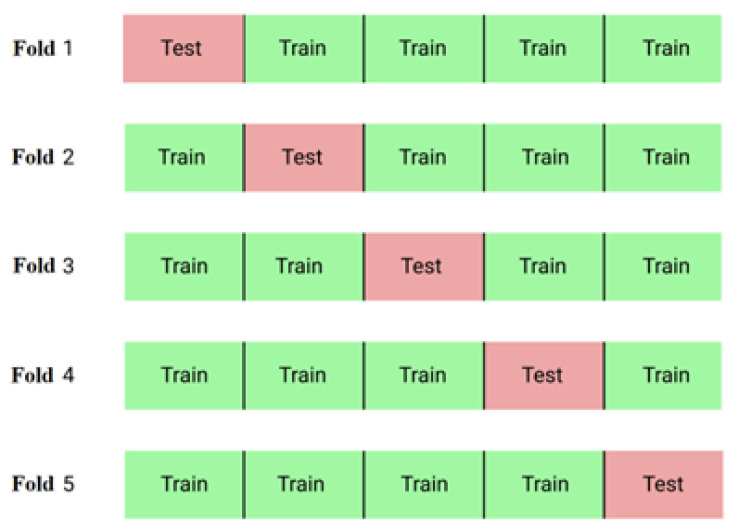
Five-fold cross-validation diagram.

(2) Deep-Learning Lung-Segmentation Test Method:

To test the deep-learning lung segmentation, we employed the 5-fold cross-validation method, as shown in Figure 7. With a total of 2000 images, these images were randomly and evenly divided into five groups, resulting in 400 images in each group. Each group of 400 images took turns serving as the testing dataset, while the remaining 1600 images were used for training. The results obtained from these five cross-validation tests were then averaged.

We used the Intersection over Union (IoU) index to assess the quality of lung-area segmentation. A detection rate was also calculated at an initial IoU threshold of 0.7 for comparison and also at various IoU thresholds. Additionally, we measured the processing time for segmentation to compare the processing speed of each method.

#### 4.1.2. Traditional Lung-Segmentation Results

**Figure 8:**
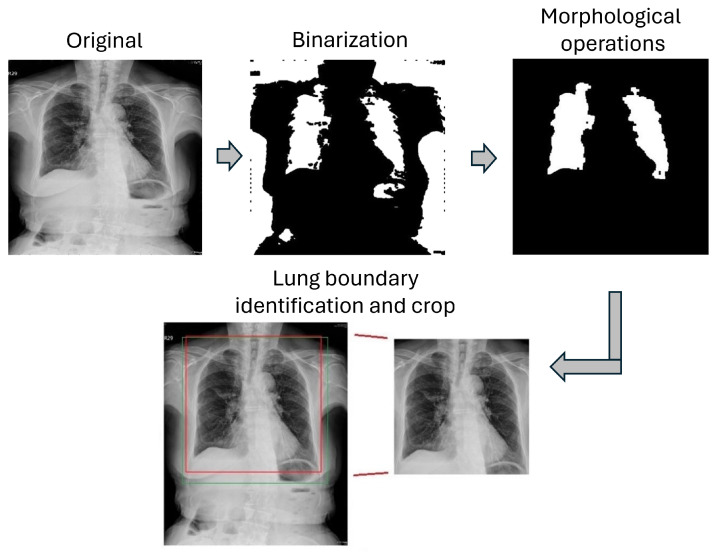
Traditional segmentation result.

The results of the traditional lung-area segmentation method at each stage of the operations are depicted in Figure 8. In the last lung boundary identification stage, the red frame indicates the detected area, while the green frame shows the ground truth area for comparison. As can be seen, while the traditional method successfully identified the general lung area, it failed to capture the finer details at the lung boundary, resulting in slight misalignment with the ground truth area.

#### 4.1.3. Deep-Learning Lung-Segmentation Results

**Figure 9:**
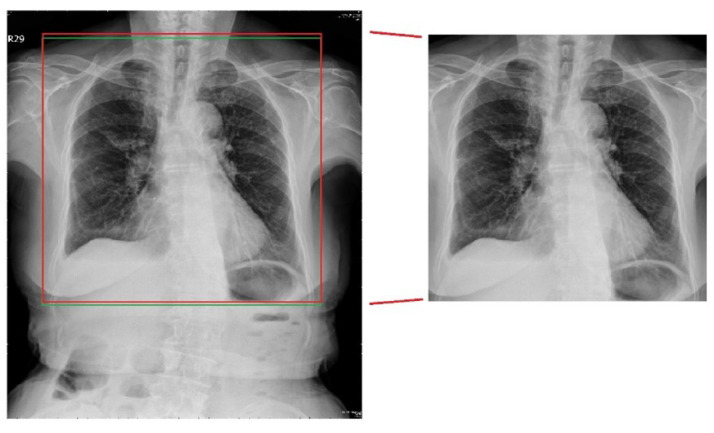
Deep-learning segmentation result.

The results of lung-area detection using the deep-learning network are depicted in Figure 9. The red frame represents the detected area, while the green frame corresponds to the ground truth area for comparison. Compared with the traditional method, the deep-learning network performs better, especially in capturing the finer details at the lung boundary, resulting in better alignment with the ground truth area.

#### 4.1.4. Comparison between Traditional and Deep-Learning Methods

Table 6 and Table 7 present a summary of the 5-fold cross-validation results, highlighting the average IoU, detection rates at an IoU threshold of 0.7, and the time taken for testing per image. These results are separately presented for both the traditional and deep learning (RetinaNet) lung-area segmentation methods.

The results clearly indicate that RetinaNet outperforms the traditional approach across all three metrics. RetinaNet achieves a notably higher average IoU of 0.963, in stark contrast to the traditional method’s IoU value of 0.833. When using an IoU threshold of 0.7, RetinaNet achieves a flawless 100.0% detection rate, while the traditional method lags behind with a detection rate of 87.65%. Additionally, the evaluation time for RetinaNet, at 0.102 s per image, is less than half the time required by the traditional approach, which takes 0.219 s per image.

**Figure 10:**
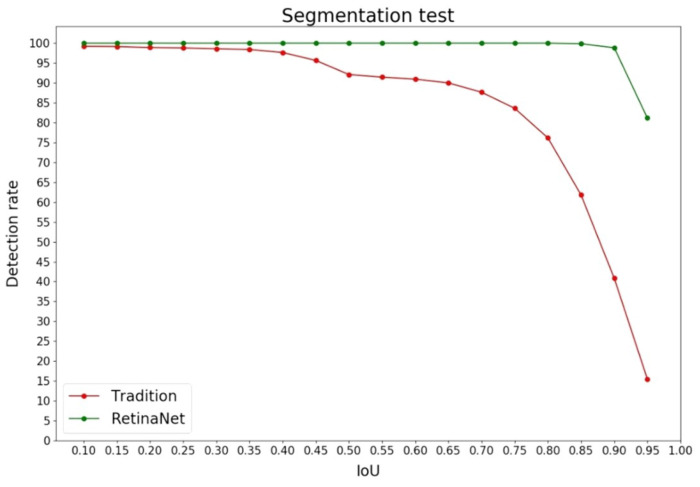
Comparison of detection rates at different IoU thresholds.

We further present the change in detection rate with respect to different IoU thresholds in Figure 10. As expected, the detection rate decreases as the IoU threshold increases. Notably, the curve for the traditional method exhibits a sharp turning point at an IoU threshold of 0.7. Conversely, RetinaNet’s turning point appears at a higher IoU threshold of 0.9. Moreover, at any given IoU threshold, RetinaNet consistently outperforms the traditional approach in terms of detection rate.

Accordingly, we employed the lung-area segmentation results obtained through RetinaNet for subsequent processing.

### 4.2. Lung Registration Experiments and Results

In the next stage, we proceed to compare the results of traditional and deep-learning lung registration methods.

#### 4.2.1. Evaluation Methods

To assess the effectiveness of lung-area registration, three quantitative indicators were utilized. The first indicator is the peak signal-to-noise ratio (PSNR) [53], which is commonly employed to evaluate the differences between processed and original images. Higher PSNR values indicate lower distortion. The second indicator is the structural similarity index measure (SSIM) [54], which measures the similarity between two images and aligns more closely with human visual judgment compared to PSNR. SSIM values range from −1 to 1, with values closer to 1 indicating greater similarity. The third indicator is the normalized cross-correlation (NCC) [55], also known as Pearson’s coefficient, which quantifies the correlation between two measures and is used to assess the correlation between image pairs. NCC values range from −1 to 1, with values closer to 1 indicating stronger correlation.

#### 4.2.2. Traditional Lung Registration Results

**Figure 11:**
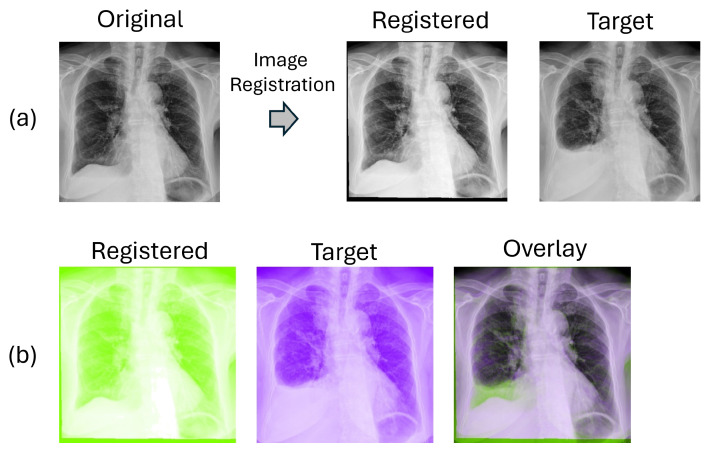
Traditional registration result. (**a**) shows the output image after registration, while (**b**) presents the comparison between the registered image and target in complementary colors.

Figure 11 presents the outcomes of the traditional lung-area registration process. Figure 11a shows the output image after registration. In Figure 11b, we present the comparison between the registered pre-image and the target post-image in complementary colors. Overlapping structures appear as black-and-white regions while differing areas are shown in color.

#### 4.2.3. Deep-Learning Lung Registration Results

**Figure 12:**
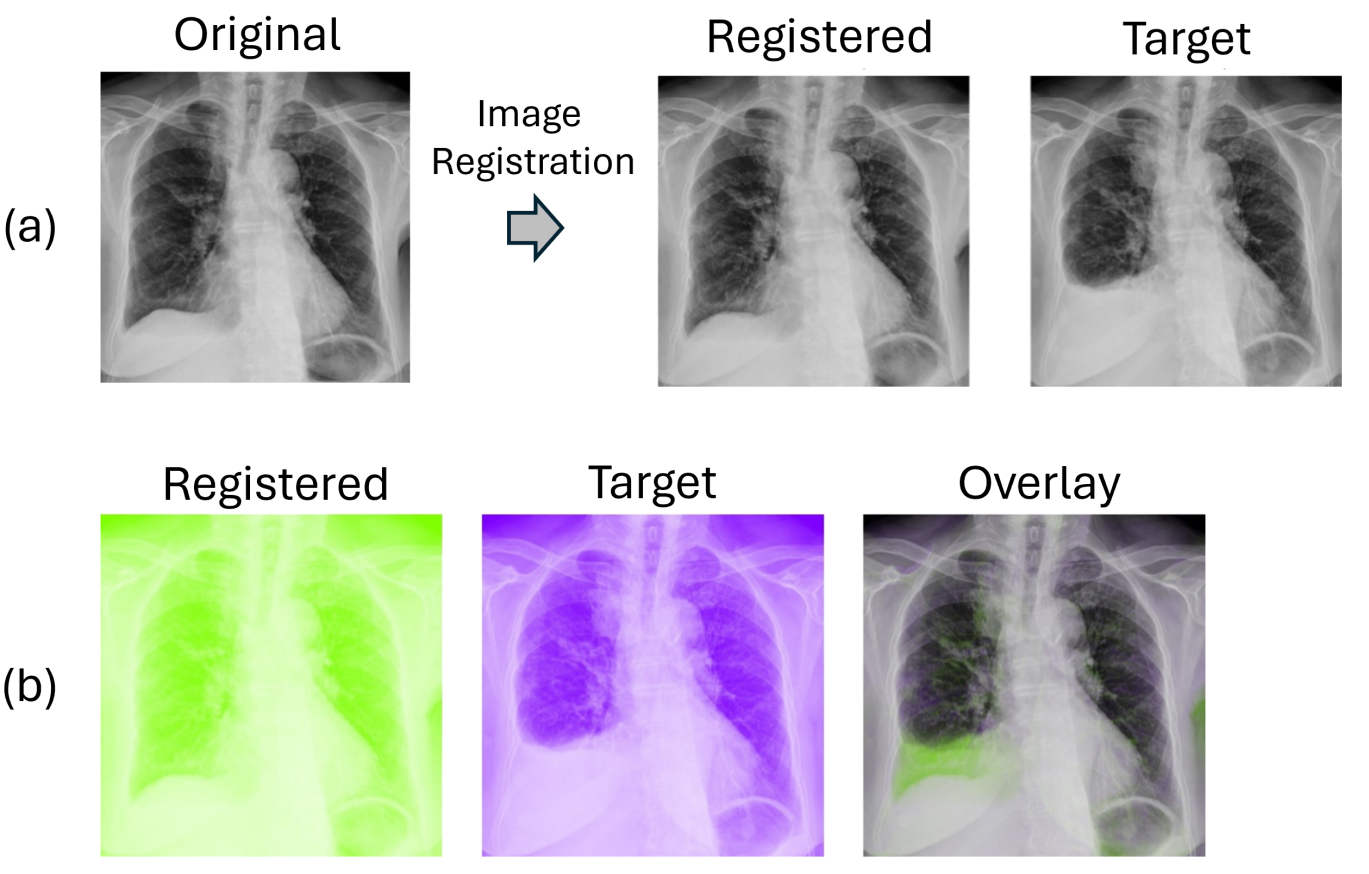
Deep-learning registration result. (**a**) shows the output image after registration, while (**b**) presents the comparison between the registered image and target in complementary colors.

Figure 12 showcases the results of the lung-area registration process using the deep-learning network VoxelMorph. Figure 12a presents the output image after registration. Similar to the traditional method, a comparative study with complementary colors is displayed in Figure 12b. It is worth noting that VoxelMorph delivers significantly improved registration results, aligning most structures and textures from the moving image with the fixed image, resulting in largely black-and-white regions. Differences shown in colors are largely not related to alignment artifacts. In contrast, the traditional registration method shows purplish color in many parts of the combined plot.

#### 4.2.4. Comparison between Traditional and Deep-Learning Methods

Two sets of testing images were used to evaluate the performance of the two registration methods. The first set consisted of 30 pairs of pre- and post-images sourced from the NIH database, distinct from the 3200 training pairs. The second set included 30 pairs of pre- and post-images from Chiayi Christian Hospital (CYCH). We calculated the average values of the three indices and the processing time for these datasets and present them in Table 8 and Table 9.

The results presented in Table 8 and Table 9 underscore the effectiveness of the two registration methods. Comparatively, the deep-learning approach using the VoxelMorph network outperforms the traditional approach employing the SimpleITK toolkit in all three registration indices while also exhibiting a significantly shorter processing time. Remarkably, the VoxelMorph network not only provided superior registration results but also achieved efficiency in terms of processing speed.

It is worth noting that the deep-learning model was trained using only data from the NIH database; thus, results on the CYCH dataset constitute a cross-dataset experiment. The promising result obtained on the CYCH dataset indicates that the model generalizes well across datasets.

Given the superior performance of the deep-learning approach, we adopted the VoxelMorph network for lung-area registration and continue with the subsequent phases of our study.

### 4.3. Lung Comparison Experiments and Results

**Figure 13:**
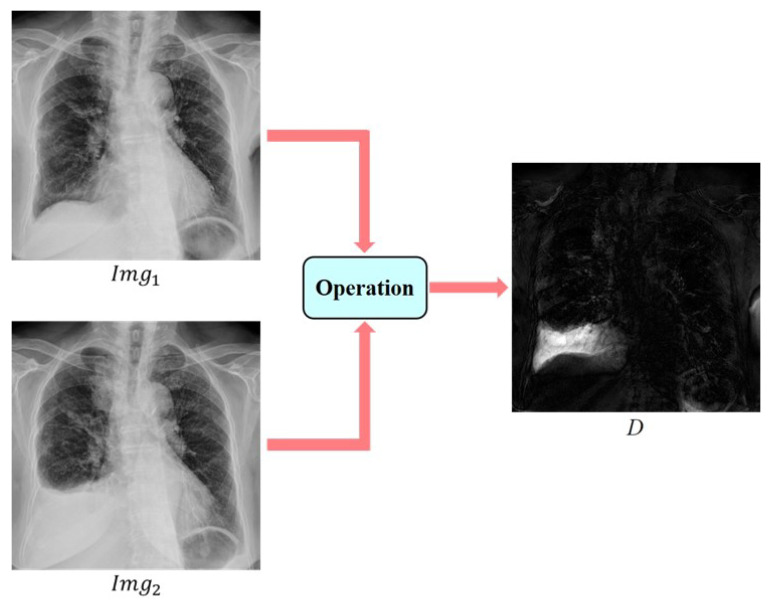
Schematic diagram for calculating the difference image.

Incorporating deep-learning approaches in both the lung-area segmentation and registration stages, we generated the difference image using specific functions to highlight distinct areas of change. Figure 13 displays a pair of pre-image (Image 1) and post-image (Image 2) selected from the CYCH dataset, along with the resulting difference image (D).

**Figure 14:**
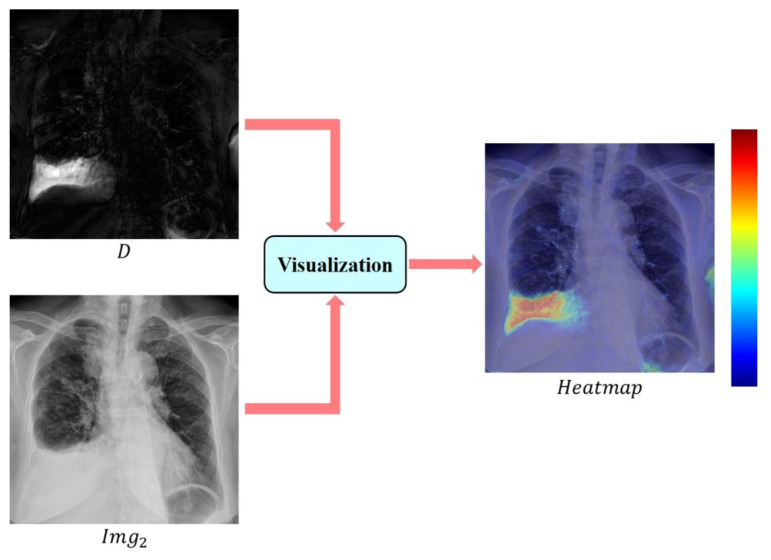
Visualized heat map.

To emphasize the changes within the difference image for human perception, we merged the information from the difference image (D) with the post-image (Image 2) and utilized a heatmap to visualize the results, as illustrated in Figure 14. The level bar indicates that redder areas represent more significant differences, while bluer areas signify smaller differences.

To enhance the visualization of the difference images, we employed three distinct display modes to transform the difference map into a heatmap. The first mode involved simply scaling the post-image with the absolute difference between the pre- and post-images, as described in Equation (1) earlier. The second and third modes were referred to as “nonlinear-1” and “nonlinear-2”, respectively. These modes used nonlinear functions, described by Equations (2) and (3), to process the absolute difference between the pre- and post-images, therefore amplifying the differences. It is worth noting that “nonlinear-2” is a more pronounced amplifier compared to “nonlinear-1.”

**Figure 15:**
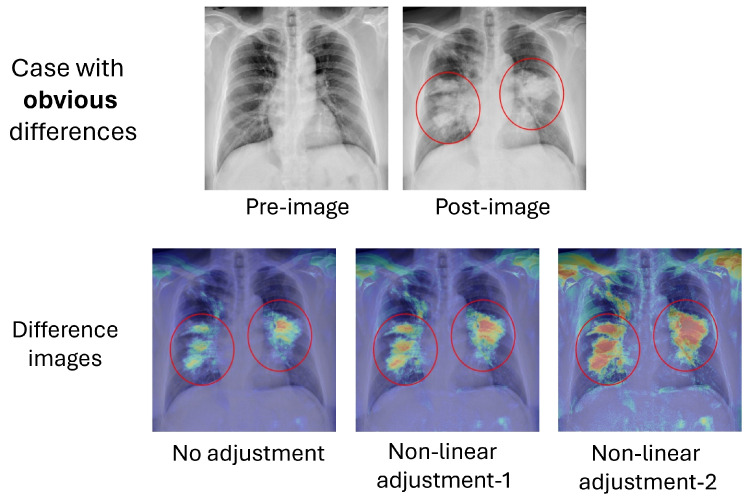
Difference images using different adjustments for the case with obvious differences. Red circles indicate areas with pathological features in the post-image.

**Figure 16:**
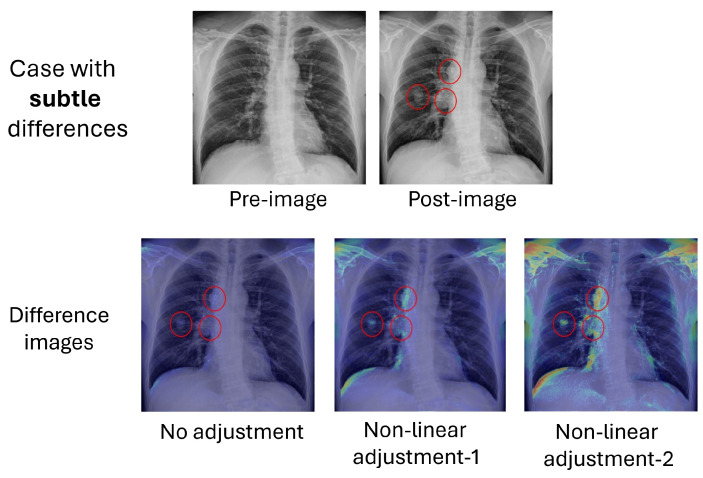
Difference images using different adjustments for the case with subtle differences. Red circles indicate areas with pathological features in the post-image.

The processing results of the three display modes are presented in Figure 15 and Figure 16 for the cases with obvious and subtle differences, respectively. It is evident that the distinctive features are emphasized at weak, medium, and strong levels through the application of different adjustments. Users can choose the appropriate mode to inspect obvious or subtle features in the heatmaps. In cases where the features are minor and less apparent, as illustrated in Figure 16, examining the heatmaps at different levels becomes crucial for identifying these distinctive features, especially in their early stages. However, it is important to note that these processes may also amplify alignment artifacts not related to disease progression, such as the clavicle and lung edge. Nevertheless, users may easily exclude these areas from consideration when assessing potential pathological features, drawing upon their anatomical knowledge to make informed judgments.

### 4.4. Lung Disease Classification Experiments and Results

In this section, we present the results of deep-learning classification experiments conducted to assess the effectiveness of using both post-images and difference image information in distinguishing different lung illnesses.

The experimental images were selected from the aforementioned NIH database, totaling 3188 pairs of pre- and post-images. Following lung image segmentation, registration, and comparison processing, these images were used as inputs for a series of chest X-ray image classification experiments. This is a multi-label task, meaning that each sample may carry more than one lung disease category. A sample that carries no lung disease labels will be in the “no findings” category. Of these, 80% of the data pairs were designated for training, with the remaining 20% reserved for testing.

#### 4.4.1. Evaluation Methods

For evaluating the performance of the lung disease classification, the area under the curve of the receiver operating characteristic curve (AUC) [56] was employed as the key evaluation index. The receiver operating characteristic curve (ROC) is a graphical tool used to assess and compare the performance of models or to determine the optimal threshold for a given model. AUC represents the area under the ROC curve, and a larger AUC value signifies better classifier performance. In this study, a 5-fold cross-validation method was employed, and the average AUC across the 5 folds was computed as the final performance metric for the classifier.

#### 4.4.2. Deep-Learning Lung Disease Classification Results

The results were categorized based on the application of single-input or dual-input networks and the three different image adjustment modes employed. Table 10 summarizes the classification results for comparison. The result when employing the single-input network and using the post-image as the sole input was considered to be the baseline for comparison. We were unable to present comparative results with other studies as none have adopted the difference image approach, and thus, the subset of the NIH dataset used in this study is unique.

For the single-input network, using the absolute difference image resulted in a 1% increase in the average AUC. The nonlinear-1 input contributed to a slightly lower increase of 0.7%, while the stronger nonlinear-2 input led to a minor decrease of −0.36% in AUC when compared to the baseline.

In the case of the dual-input network, the addition of different image information significantly improved classification accuracies in all three adjustment modes. In summary, the dual-input networks outperformed the single-input networks. This observation demonstrates the capability of providing both the original image and the difference image in enhancing the average AUC. Comparatively, the second input image contributed to 1.21%, 1.31%, and 1.39% increases in the average AUC compared to the baseline when using linear, nonlinear-1, and nonlinear-2 adjustments, respectively. The strength of the adjustment was shown to influence the extent of improvement in the average AUC.

The inclusion of difference image information in the network inputs indeed affected the classification results of both the single- and dual-input networks but in different ways. Embedding the difference image information with linear adjustment increased the average AUC in both network architectures. However, the two nonlinear adjustment modes influenced the two architectures differently. The single-input network responded to the adjustment inversely to the level of difference amplification, i.e., a decrease in the average AUC with increased amplification. In contrast, the dual-input network responded positively to the adjustment, resulting in an increase in the average AUC with the level of amplification. This suggests that the adjustments highlight the difference areas between the pre- and post-images but also alter the original images. In a single-input network, this alteration emphasized distinctive features with weak (linear) and medium (nonlinear-1) adjustments but may distort key features with very strong (nonlinear-2) adjustments, leading to an adverse effect. In contrast, the adjustments added information to the dual-input network as a second input. The strength of adjustment, whether weak, medium, or strong, all contributed to providing additional information through the second input, and the strength of the adjustment contributed to improving disease differentiation (classification) to a certain extent.

## 5. Conclusions

This research intended to develop a chest X-ray imaging system to assist physicians in the diagnosis and progression analysis of chest-related diseases, such that the efficiency can be improved and misjudgments reduced. In this study, the system is divided into four stages, namely lung image segmentation, registration, comparison, and classification stages. A computer-aided diagnosis system based on deep learning was proposed, and its efficacy was compared to that of the traditional approach.

In the lung image segmentation stage, the deep-learning approach, which uses RetinaNet to detect the lung area, outperforms the traditional image-processing approach. The segmentation performances of the two methods were evaluated using Intersection over Union (IoU), detection rate, and evaluation time and are validated using 5-fold cross-validation. The results showed that the deep-learning approach outperformed the traditional approach in all three metrics, and the average evaluation time of the deep-learning approach is only about half of that of the traditional approach. Moreover, when tested with different IoU thresholds, the detection rate of the deep-learning network maintains at 100% through threshold ranges from 0.1 to 0.85, always higher than the traditional method. This observation highlights the superiority of the deep-learning approach over the traditional one in lung-area segmentation.

In the lung image registration stage, this study compared a deep-learning approach VoxelMorph to the traditional approach based on the open-source software SimpleITK toolkit [47] . Evaluation metrics, including the peak signal-to-noise ratio (PSNR), structural similarity (SSIM), normalized cross-correlation (NCC), and evaluation time, were used to evaluate the performance in lung-area image registration by the two approaches. The results showed that both approaches achieved outstanding performance in registration. However, it is noted that the deep-learning approach obtained better results based on the three metrics, especially in the evaluation time, where the deep-learning approach only takes 1/18 the time as the traditional approach.

In the lung image comparison stage, the differences between the pre- and post-images were amplified using nonlinear transformations and presented as a heat map using the colormap library provided by OpenCV. Different nonlinear transformations can be chosen by users to amplify the differences between the two images.

In the lung image classification stage, two DenseNet architectures, one with single input and the other with dual inputs, were proposed, and their performance was compared. The area under the curve (AUC) index was employed to evaluate the effectiveness of the classifiers using the 5-fold validation method. The performance of the single-input DenseNet using the post-image was set as the baseline for comparison. Using the single-input DenseNet, a slightly higher average AUC (1% increase) was observed in the experiment using a simple difference image as input when compared to that of the baseline. However, over-adjusting the difference information using the nonlinear transformations may otherwise reduce the average AUC. As for the dual-input DenseNet, different combinations of the two input images all yielded a higher AUC (about 1.2∼1.4% increase) when compared with that of the baseline. These observations confirm the effectiveness of the additional difference information in raising the recognition capability of the classifier in lung disease identification.

However, limited by the lack of a dataset that contains image pairs from the same patient taken at different times, the achieved AUC is still far from satisfactory for actual clinical application. Nevertheless, the results of this study demonstrate the potential of deep learning in the comparative analysis of chest X-ray images. 

## Figures and Tables

**Table 1:** The 3188 pairs of images for classification.

Disease	Training	Testing	Sub-Total
Atelectasis	190	48	238
Cardiomegaly	49	12	61
Effusion	176	44	220
Infiltration	579	145	724
Mass	65	16	81
Nodule	202	50	252
Pneumonia	32	8	40
Pneumothorax	102	25	127
Consolidation	45	11	56
Edema	0	0	0
Emphysema	30	7	37
Fibrosis	70	17	87
Pleural Thickening	86	22	108
Hernia	0	0	0
No Finding	1280	320	1600
Total	2906	725	3631

**Table 2:** Hyperparameter settings for the lung detection network.

Hyperparameters	Value
Batch size	1
Learning rate initial value	0.0001
Learning rate decay factor	0.1
Patience	3
Epochs	200
Optimizer	Adam (betas = (0.9, 0.999))

**Table 3:** Hyperparameter settings for the lung registration network.

Hyperparameters	Value
Batch size	8
Learning rate initial value	0.0001
Epochs	400
Optimizer	Adam (betas = (0.9, 0.999))

**Table 4:** Hyperparameter settings for the classification model.

Hyperparameters	Value
Batch size	2
Learning rate initial value	0.0001
Learning rate decay factor	0.1
Patience	3
Epoch number	40
Optimizer	Adam (betas = (0.9, 0.999))

**Table 5:** Hardware specifications.

Hardware	Specifications
CPU	Intel Xeon W-2125 (Quadcore) 4.0 GHz
Motherboard	ASUS WS880T
RAM	16 GB DDR4-2666 RDIMM.ECC
HDD	WD WD20EARS-00MVWB0 2 TB
GPU	NVIDIA GeForce RTX 2080 Ti 11 GB

**Table 6:** Traditional segmentation performance result.

Traditional	Fold-1	Fold-2	Fold-3	Fold-4	Fold-5	Avg
Avg IoU	0.841	0.836	0.838	0.821	0.827	0.833
Detection rate (%) (IoU = 0.7)	89.25	88.50	87.50	85.75	87.25	87.65
Evaluation time (s/image)	0.221	0.219	0.217	0.220	0.220	0.219

**Table 7:** Deep-learning segmentation performance result.

RetinaNet	Fold-1	Fold-2	Fold-3	Fold-4	Fold-5	Avg
Avg IoU	0.964	0.963	0.962	0.962	0.964	0.963
Detection rate (%) (IoU = 0.7)	100.0	100.0	100.0	100.0	100.0	100.0
Evaluation time (s/image)	0.102	0.101	0.102	0.103	0.101	0.102

**Table 8:** Lung registration result on NIH dataset.

Metrics (Mean)	Without Registration	Traditional (SimpleITK)	Deep Learning (VoxelMorph)
PSNR (dB)	26.6321	28.6785	29.1430
SSIM	0.7706	0.8538	0.8679
NCC	0.8827	0.9276	0.9286
Evaluation time (s/image)	-	37.633	1.925

**Table 9:** Lung registration result on CYCH dataset.

Metrics (Mean)	Without Registration	Traditional (SimpleITK)	Deep Learning (VoxelMorph)
PSNR (dB)	20.6571	23.1931	23.3901
SSIM	0.5930	0.7151	0.7697
NCC	0.8636	0.9198	0.9207
Evaluation time (s/image)	-	37.371	2.041

**Table 10:** Comparison of AUC results for each architecture and input modes.

Network Architecture	Single-Input DenseNet [51] (Baseline)	Single-Input	Single-Input	Single-Input	Dual-Input	Dual-Input	Dual-Input
**Input Type**	**Original Image**	**Linear Difference Image**	**Nonlinear-1 Difference Image**	**Nonlinear-2 Difference Image**	**Original Image + Linear Difference Image**	**Original Image + Nonlinear-1 Difference Image**	**Original Image + Nonlinear-2 Difference Image**
Atelectasis	0.7691	0.7644	0.7414	0.7293	0.7573	0.7676	0.7482
Cardiomegaly	0.8412	0.8384	0.7945	0.7813	0.7867	0.8043	0.8547
Effusion	0.8414	0.8547	0.8567	0.8325	0.8422	0.837	0.8387
Infiltration	0.5553	0.5754	0.5745	0.5419	0.5714	0.5604	0.5435
Mass	0.5605	0.5868	0.6087	0.5866	0.5880	0.6162	0.5743
Nodule	0.5530	0.5456	0.5415	0.5501	0.5614	0.5641	0.5547
Pneumonia	0.6251	0.6132	0.6223	0.6673	0.6271	0.5987	0.6257
Pneumothorax	0.7768	0.7981	0.7886	0.7767	0.8161	0.8097	0.8055
Consolidation	0.6686	0.6614	0.6795	0.677	0.7195	0.7203	0.7190
Emphysema	0.6627	0.7247	0.735	0.6783	0.6721	0.7094	0.6824
Fibrosis	0.6367	0.6525	0.6302	0.6414	0.6764	0.6559	0.6883
Pleural Thickening	0.6902	0.6854	0.692	0.6744	0.7075	0.6939	0.7117
Mean	0.6817	0.6917	0.6887	0.6781	0.6938	0.6948	0.6956

## Data Availability

The TCGA dataset is publicly available, and can be found here: https://www.kaggle.com/datasets/nih-chest-xrays/data (accessed on 1 January 2020). The CYCH dataset is not publicly available due to privacy reasons.

## Abbreviations

The following abbreviations are used in this manuscript:

NIH	National Institutes of Health
CYCH	Chiayi Christian Hospital
ROI	Region of interest
CLAHE	Contrast Limited Adaptive Histogram Equalization
CNN	Convolutional neural networks
ResNet	Residual Network
FPN	Feature Pyramid Network
GAP	Global Average Pooling
ANTS	Advanced Normalization Tools
IoU	Intersection over Union
PSNR	Peak signal-to-noise ratio
SSIM	Structural similarity index measure
NCC	Normalized cross-correlation
ROC	Receiver operating characteristic curve
AUC	Area under the ROC curve
